# Automatic processing of unattended lexical information in visual oddball presentation: neurophysiological evidence

**DOI:** 10.3389/fnhum.2013.00421

**Published:** 2013-08-09

**Authors:** Yury Shtyrov, Galina Goryainova, Sergei Tugin, Alexey Ossadtchi, Anna Shestakova

**Affiliations:** ^1^Center of Functionally Integrative Neuroscience, Institute for Clinical Medicine, Aarhus UniversityAarhus, Denmark; ^2^Centre for Languages and Literature, Lund UniversityLund, Sweden; ^3^Medical Research Council, Cognition and Brain Sciences UnitCambridge, UK; ^4^Department of Higher Nervous Activity and Psychophysiology, Saint Petersburg State UniversitySaint Petersburg, Russia; ^5^Department of Biomedical Engineering and Computational Science, Aalto UniversityEspoo, Finland; ^6^Institute for Problems of Mechanical Engineering, Russian Academy of SciencesSt. Petersburg, Russia; ^7^MEG Centre, Moscow State University of Psychology and EducationMoscow, Russia

**Keywords:** brain, language, event-related potential (ERP), mismatch negativity (MMN, vMMN), lexical memory trace, visual word comprehension

## Abstract

Previous electrophysiological studies of automatic language processing revealed early (100–200 ms) reflections of access to lexical characteristics of speech signal using the so-called mismatch negativity (MMN), a negative ERP deflection elicited by infrequent irregularities in unattended repetitive auditory stimulation. In those studies, lexical processing of spoken stimuli became manifest as an enhanced ERP in response to unattended real words, as opposed to phonologically matched but meaningless pseudoword stimuli. This lexical ERP enhancement was explained by automatic activation of word memory traces realized as distributed strongly intra-connected neuronal circuits, whose robustness guarantees memory trace activation even in the absence of attention on spoken input. Such an account would predict the automatic activation of these memory traces upon any presentation of linguistic information, irrespective of the presentation modality. As previous lexical MMN studies exclusively used auditory stimulation, we here adapted the lexical MMN paradigm to investigate early automatic lexical effects in the visual modality. In a visual oddball sequence, matched short word and pseudoword stimuli were presented tachistoscopically in perifoveal area outside the visual focus of attention, as the subjects' attention was concentrated on a concurrent non-linguistic visual dual task in the center of the screen. Using EEG, we found a visual analogue of the lexical ERP enhancement effect, with unattended written words producing larger brain response amplitudes than matched pseudowords, starting at ~100 ms. Furthermore, we also found significant visual MMN, reported here for the first time for unattended perifoveal lexical stimuli. The data suggest early automatic lexical processing of visually presented language which commences rapidly and can take place outside the focus of attention.

## Introduction

In spite of years of productive research in psycho- and neuro-linguistics as well as psychophysiology and cognitive neuroscience, neurobiological mechanisms underlying the human language function remain poorly understood. Some of the questions still hotly debated in language sciences are the time course of linguistic processes in the brain and the degree of their dependence on attentional control. When exactly are word representations assessed by the brain? How automatic is this process and/or does it require our conscious control? While some scientists have traditionally argued for a lexico-semantic access at 350–400 ms (see e.g., Friederici, 2002; Hagoort, 2008), some more recent evidence is pointing toward a much earlier onset of these processes, at ~50–200 ms (Pulvermüller et al., 2009; MacGregor et al., 2012). Similarly, whereas some accounts of linguistic processes imply attentional control over them, there are strong indications of a large degree of automaticity in e.g., lexico-semantic and syntactic processes, at least at their earliest stages (for a review, see e.g., Shtyrov, 2010).

A substantial contribution to this debate came from a body of recent investigations using non-attend designs, where the subjects are not given a stimulus-related task and, furthermore, are distracted from auditory linguistic stimuli by an alternative primary task. This is done in order to ensure that no interference can come from attentional biases and stimulus-specific behavioral strategies^1^. A large number of these studies have used the so-called mismatch negativity (MMN) brain response, an early component of auditory event-related potentials (ERPs). MMN shows high sensitivity to unexpected changes in a monotonous stream of unattended sounds, reflected in electroencephalographic (EEG) recordings as an increased fronto-central negativity with temporo-frontal sources (Näätänen et al., 2007). When these sounds are meaningful speech elements, for example words or morphemes of a native language, they show a characteristic ERP amplitude increase over acoustically similar and psycholinguistically matched stimuli that do not form meaningful language units. Dubbed “lexical enhancement,” this phenomenon, which most often occurs at about 100–200 ms, has been investigated in different experimental settings, languages and imaging modalities (EEG, MEG, fMRI; see e.g., Korpilahti et al., 2001; Shtyrov and Pulvermüller, 2002; Shtyrov et al., 2005, 2008). This word-specific brain response shows sensitivity to a number of psycholinguistic word properties: its amplitude changes with word frequency (Alexandrov et al., 2011; Shtyrov et al., 2011), its surface topography and underlying cortical sources show specificity to word semantics (Shtyrov et al., 2004; Pulvermüller et al., 2005), its latency correlates with psycholinguistically determined word recognition times (Pulvermüller et al., 2006), etc. This has led to firm conclusions that lexical MMN response reflects activation of neural memory traces for stimulus words, which occurs rapidly after the information at the auditory input allows for word identification (Pulvermüller and Shtyrov, 2006). Importantly, this activation takes place when the subjects' attention is removed from the linguistic stimuli. Furthermore, modulation of attention levels (using task demands and experimental instructions) does not affect the strength of this early word-elicited response (Garagnani et al., 2009; Shtyrov et al., 2010). These latter findings imply that the early word-specific activation is largely automatic and does not strongly depend on the level of attentional control. This automaticity could be attributed to the robustness of distributed neuronal networks that act as neural word memory traces in the brain. Importantly, these findings of early automatic lexical activation could also be replicated outside the MMN oddball paradigm, in an ecologically more valid presentation of multiple unrepeated words and pseudowords, provided their acoustic and phonological features are tightly controlled (MacGregor et al., 2012). In sum, this body of evidence suggests that the brain may be capable of automatic lexical analysis of spoken language even in the absence of attention on the linguistic input.

Such an account would predict the automatic activation of these memory traces upon *any* presentation of linguistic information, irrespective of the modality in which it is presented. To date, however, linguistic experiments in the visual modality have not been able to explore this phenomenon, as they have usually presented stimuli in the focus of attention. In terms of the speed of lexico-semanitc activation, a number of visual studies provide a similar picture of rapid and early access to word information in the brain, as seen in visual ERPs at latencies between 100–200 ms (e.g., Ortigue et al., 2004; Hauk et al., 2006). Such studies, however, cannot easily address the question of automaticity of neural lexical access. Indeed, it is not easily possible to present *unattended* words visually: if the stimulus falls within the focus of the visual field, it enters the attended area, which is why visual research mostly deals with active processing of attended stimuli. One approach to study subconscious visual word processing is masked priming (e.g., Dehaene et al., 2001; Henson, 2003) where a “probe” word may be preceded by a “prime” stimulus, which is masked and presented so briefly that the subject is not able to consciously register it. Masked priming studies have indeed reported a number of effects produced by such “invisible” word stimuli, including evidence of lexico-semantic access to them (e.g., Brown and Hagoort, 1993; Kiefer, 2002), although at later latencies than in the auditory studies above. However, such experiments, on the one hand, *do* require vigilant attention to the linguistic input (and thus rather reduce awareness than remove attention). On the other hand, priming studies (masked priming included) more likely assess the interactions between the prime and the probe rather than the processing of the subliminal stimulus *per se*. A similar comment can be made with respect to the visual Stroop task which famously demonstrated behaviorally (e.g., Glaser and Glaser, 1989) the automaticity in access of individual words^2^ : whilst the experimental instruction *per se* does not explicitly encourage word processing, the stimulus words themselves in the Stroop task are nevertheless presented in the focus of attention. Thus, the automaticity of neural processing of *unattended* visual language remains obscure.

To complement the earlier auditory MMN studies and bridge the gap between them and the visual modality in linguistic processing, we set out to address the issue of early lexical automaticity in the visual domain. For this, it seems essential to remove the focus from the visual linguistic input (similar to the previous auditory research above) and to record activations caused by unattended stimuli *per se*. For maximum compatibility with the previous research, we decided to adapt the auditory lexical MMN paradigm to the visual modality. A visual analogue of the auditory MMN (vMMN) is known to occur for presentation of at least non-linguistic graphical stimuli (Czigler et al., 2006). This usually involves a primary task such as tracking geometrical shapes in the center of the visual field, while unattended stimuli (frequent “standards” and rare unexpected “deviants”) are flashed on the periphery of the visual field in oddball sequences, similar to those used auditorily. vMMN can be elicited independently of attention (Berti, 2011) by deviance in color (Czigler et al., 2002), orientation (Astikainen and Hietanen, 2009; Kimura et al., 2010), movement (Pazo-Alvarez et al., 2003), spatial frequency (Heslenfeld, 2003), contrast (Stagg et al., 2004) and even in abstract sequential regularities (e.g., “if, then … ” rules; Stefanics et al., 2011) in visual stimulation. Whilst having been linked to neural automatic visual change detection and short-term memory (Czigler and Pato, 2009), vMMN has remained virtually unexplored with respect to its sensitivity to long-term representations, such as word-specific lexical memory circuits.

Motivations for applying MMN methodology to language lie, on the one hand, with the earliness and automaticity of this cognitive ERP (Shtyrov and Pulvermüller, 2007). These properties make it instrumental for uncovering the earliest attention-independent neurophysiological indices of language processing, without any confounds associated with active tasks and attention variation (Pettigrew et al., 2004; Pulvermüller and Shtyrov, 2006; Näätänen et al., 2007). From the other, more methodological point of view, the use of a small set of well-controlled stimuli minimizes stimulus variance and associated brain response smearing, allowing for a finer degree of precision in locating and analysing any minute short-lived early activations (Shtyrov and Pulvermüller, 2007). Further, as the MMN is a difference response (obtained as a deviant-minus-standard ERP subtraction), this helps to rule out purely sensory confounds arising from divergence of physical stimulus features, by incorporating identical physical contrasts into different linguistic contexts. An advantage of the visual presentation, on the other hand, is its potential ability to overcome inherent problems of spoken stimulus presentation, such as variability in word length, in sound energy distribution across the waveform's duration, in word-specific recognition points etc. Unlike auditory stimuli that unfold over time, visual words are available in full instantly and can be presented for a strictly defined period of time, which can be fully matched across stimuli and conditions.

To test the presence of early automatic lexical effects in visual oddball presentation, we adapted the established lexical MMN approach to the visual modality. In line with non-linguistic visual MMN research (see e.g., Pazo-Alvarez et al., 2003), we engaged our experimental participants in a primary non-linguistic task continuously present in the center of the visual field. While the subjects were focused on this primary task, words and pseudowords matched for physical properties were briefly (100 ms) flashed just outside the fovea (2.5°) in oddball sequences. All sequences had identical single-letter visual standard-deviant contrasts, while the exact lexical status of the standard and deviant stimuli (as either words or pseudowords) was systematically modulated. To control for purely sensory effects, further non-linguistic control stimuli were used, and a low-level visual baseline condition was applied to parcel out the primary task contribution to visual responses. The subjects' neural responses to the stimulation were recorded using EEG. Based on the previous research, we expected to observe an early reflection of lexical differences, most likely as an increase in word-elicited activation relative to pseudoword ERPs. We also expected a visual MMN in the form of a difference between the deviant and standard brain responses.

## Methods

### Subjects

Sixteen healthy right-handed (handedness assessed according to Oldfield, 1971) native Russian-speaking volunteers (6 males; age range 18–24, mean 21.2 y.o.) with normal vision and no record of neurological diseases were presented with visual stimuli in 6 experimental conditions. All subjects gave their written consent to take part in the study and were paid for their participation. The experiments were performed in accordance with the Declaration of Helsinki with approval of the University of St. Petersburg Ethics Committee.

### Stimuli

#### Oddball stimuli

As linguistic stimuli in the visual oddball presentation, we employed four sets of controlled monosyllabic three-letter words and pseudowords of the Russian language (Table 1). All stimuli were closely matched in their properties: (1) the two words in each standard-deviant pair shared the first two letters (always consonant-vowel), (2) the visual/orthographic contrasts between the standard and the deviant stimuli were identical in all conditions, and comprised a change between word-final consonants “
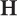
” [k]^3^ and “
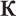
” [n], (3) the four sets differed only in the first letter (“m” [m], “t” [t], “
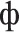
” [f], “
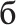
” [b], which was however the same letter within each set), (4) because of transparency in Russian orthography, the sets possessed equal phonetic similarity and identical phonetic contrasts in the auditory domain, which could be important to control in case of their covert articulation, even though it is unlikely to take place given the procedures employed (see below). All words were lexically unambiguous nouns common in Russian language and had similarly high lexical frequency of occurrence (range: 1.51–2.08 log instances per million; determined according to Sharoff, 2001), as did stimulus-initial and stimulus-final bigrams (2.27–3.18 and 3.04–3.12, respectively). Whilst matched visually and orthographically, the four sets systematically differed in the lexical status of the standard and deviant stimuli. All possible combinations were included: standard word vs. deviant word, standard pseudoword vs. deviant pseudoword, standard word vs. deviant pseudoword and standard pseudoword vs. deviant word (see Table 1).

**Table 1:**
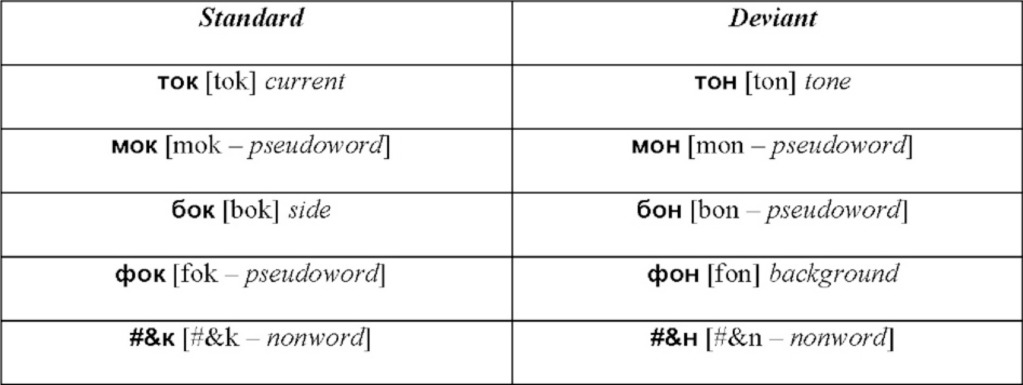
**Visual word, pseudoword and non-word stimuli used in oddball sequences (Latinized transcription in square brackets, English translation in italics)**.

*Note that the stimuli are very similar orthographically, while their lexical status is modulated systematically. Visual standard-deviant contrast* (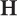/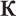 *[k/n]) is identical across all conditions.*

To validate our choice of lexical stimuli and ensure that they were perceived as meaningful words vs. meaningless pseudowords by all experimental participants, we administered a behavioral rating questionnaire to all participants (after the EEG recording). This included answering questions on stimulus lexicality (“how confident are you that this is a real word in the Russian language”) and frequency (“how often do you encounter this word or use it yourself”) on a 7-point Likert scale. This rating study fully confirmed the intended strong word-pseudoword distinction (lexicality ratings: 6.9 words vs. 1.6 pseudowords [*F*_(1, 15)_ = 692, *p* < 0.0001]; frequency rating: 6.1 vs. 1.2 [*F*_(1, 15)_ = 252, *p* < 0.0001])^4^.

In addition to the 4 word and pseudoword conditions, a non-word stimulus set was included to control for lower-level sensory/sublexical factors. To match this set with the main 4 conditions, it employed the same visual contrast (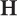/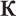) incorporated with non-orthographic symbols of hashmark and ampersand, not typically used in Russian (see Table 1).

**Figure 1:**
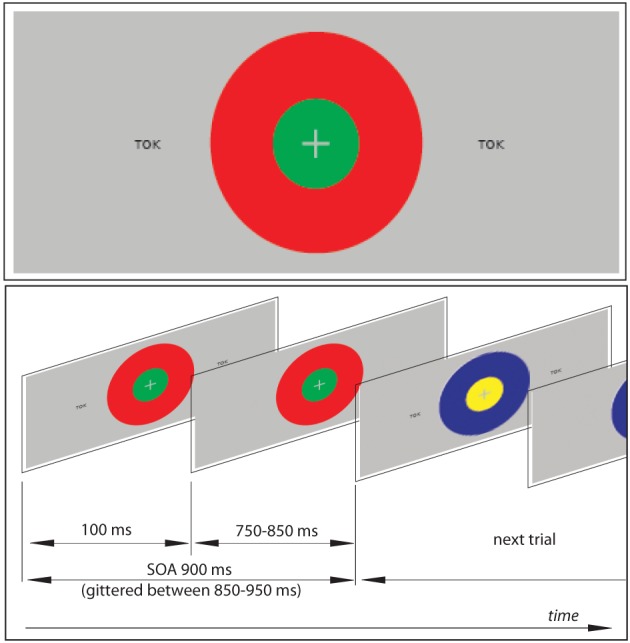
**An example of the visual stimulation employed and a schematic demonstration of the visual sequence.** The subjects' task was to focus on the center of the screen to detect combinations of two concentric circles, which were present continuously but changed colors pseudorandomly at every SOA refresh. At the same time, unattended orthographic stimuli were presented briefly (100 ms) at symmetrical locations on visual periphery (at 2.5° angle from the center to the left and to the right) in oddball sequences containing frequent standard and rare unexpected deviant stimuli (see also Table 1). In addition to the set of oddball blocks, a sensory visual baseline condition was included that only contained concentric circles but no orthogprahic stimuli on the flanks.

The textual stimuli were presented tachistoscopically for 100 ms, with stimulus onset asynchrony jittered between 800–1000 ms (mean 900 ms), in black font-face (Arial 14 pt) on grey background (Figure 1). Two copies of each stimulus were simultaneously displayed at symmetric locations in the left and right hemifields at 2.5° angle from the center of the screen. Such a symmetric bilateral presentation was used in order to ensure that, while the complete information is presented to both visual hemifields, the participant's gaze is not prompted to saccade from the central task to the orthographic stimuli (the risk of which could be higher with a single asymmetric presentation).

#### Non-linguistic primary task stimuli

As a primary task, which the participants were instructed to concentrate on, they were presented with 2 concentric circles of different colors (Figure 1): all possible combinations of red, green, blue and yellow were used. These combinations were displayed in the center of the screen and changed in synchrony with the orthographic stimuli that appeared on visual periphery. However, unlike the latter, these were kept on the screen for the entire duration of the SOA (to avoid strong visual onset and offset responses) such that the circles were seen as present continuously, with their colors changing.

## Procedure

The subjects were instructed to fixate their gaze on the center of the screen where a fixation cross was displayed, and to focus on a dual visual task of detecting color circle combinations presented in the focus of their visual attention. This dual task required tracing the color of both the inner and the outer circles and reacting only to a particular combination of colors/locations (i.e., when the task was to detect “inner red, outer blue” target, responses to any other combination—including “inner blue, outer red”—were considered incorrect). Responses were given by pressing a button with the left index finger. In addition, the subjects were requested to count the number of target combinations and report them at the end of the block. Target combination probability was 15%. As the experiment consisted of six blocks, a different target combination was used in each block. The order of target color combinations was counterbalanced across subjects, and, within each block, stimulus sequences were randomized individually. A short training sequence, using similar (but not identical) stimuli was run in the beginning of each experiment.

While the subjects concentrated on this primary task, unattended orthographic stimuli were presented at the flanks. Each standard-deviant pair was presented in a separate block, where 600 frequent standard stimuli were pseudo-randomly interspersed with 100 deviant ones. There were at least two standard presentations between any two deviants. The subjects were not informed of the orthographic stimuli, and the task did not encourage attention on them. On the contrary, the very brief presentation of these stimuli (100 ms) that appeared perifoveally at the same time as the color combinations were changing in the focus of their attention ensured maximum distraction from the textual stimulation.

In addition to the four word/pseudoword sets and one non-word set, one further condition was included that contained only the primary visual detection task but no text stimuli. This was done in order to establish the baseline level of brain activation related purely to the colored geometric shapes, which could later be used to parcel out text-related brain responses from those related to the concurrent non-linguistic task.

### EEG recording and pre-processing

During the visual presentation, the subjects' EEG was registered using a 32-channel EEG setup (Mitsar, St. Petersburg, Russia) and 10-mm gold-plated electrodes (Grass Products, Warwick RI, USA) placed on the scalp according to the 10–20% electrode configuration system, with linked mastoids as a reference electrode. To control for vertical and horizontal eye movements, electrooculogram (EOG) readings were taken via two electrodes placed below the left eye and lateral to its outer canthus. The sampling rate was 500 Hz. Electrode impedances were kept below 5 kΩ.

EEG data analysis was carried out offline using EMSE Suite (Source Signal, La Mesa CA, USA). Data were re-referenced to average reference, band-pass filtered (1–30 Hz) and bipolar electro-oculogram channels were reconstructed for vertical (VEOG) and horizontal (HEOG) eye movements from monopolar EOG recordings. Continuous data were then epoched into segments starting 100 ms before stimulus onset and ending 600 ms thereafter. The prestimulus interval of −100–0 ms was used as a baseline. Any epoch with signal variation exceeding 100 μV was discarded, as were those that coincided with any target stimuli and the ones immediately following them, to minimize buttonpress-related movement artifacts. The remaining artifact-free epochs were then averaged separately for each stimulus type (standard/deviant, word/pseudoword etc.). Finally, ERPs obtained for the control primary task-only block were subtracted from those obtained in the text stimulation blocks, in order to remove any contribution of attended geometric shapes into the responses, and concentrate on the effects of unattended orthographic stimuli *per se*.

### EEG statistical analysis

For an unbiased data-driven analysis, overall activation strength of the ERPs was first quantified as the global root mean square (RMS) of the ERP responses across all scalp electrodes. To this end, the grand average response was calculated across all word and pseudoword stimuli collapsed (standards and deviants included) for each electrode. Then, for each time point, the square root was calculated on the mean of squared amplitudes across all electrodes, producing a single global RMS response. Finally, the most prominent peaks in this global RMS were identified. These were found at ~110 and 250 ms, which coincided with the well-known ERP responses to visual/written stimuli: N1/P100 and N250 (Oken et al., 1987; Carreiras et al., 2009; Lee et al., 2012). Mean amplitudes across 20-ms time windows centered on these peaks were used for a more detailed further analysis. A smaller deflection was found at ~350–400 ms corresponding to the established N350/N400 effects (Bentin et al., 1999; Lau et al., 2008); this period was therefore used as a 3rd time window in statistical assessment of the ERPs.

For statistical analysis, window-mean amplitudes extracted from each electrode in a 25-electrode array (organized in a 5 × 5 grid) covering most of the scalp were submitted to analyses of variance using factors Lexicality (words vs. pseudowords/non-words), Stimulus Type (Standard—Deviant) and Topography (electrode location). For these statistics, data were taken from ERP responses prior to the RMS procedures, in order to allow assessment of possible polarity and topography differences.

## Results

**Figure 2:**
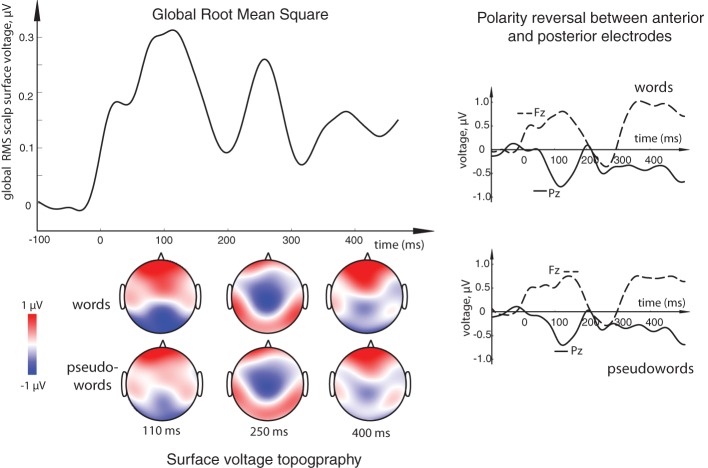
**(Top left) Overall activation elicited by the orthographic stimuli in the current task: global root mean square (RMS) calculated for all word and pseudoword responses (both standards and deviants) over all conditions, all subjects and all electrode locations.** Three distinct peaks, at ~110, ~250, and ~375 ms could be observed as the most prominent activation occurring in this general stimulus-unspecific RMS, thus determining key intervals to be later used for statistical comparisons. **(Bottom left)** Surface voltage topography of word and pseudoword ERPs at the three main peaks. **(Right)** Examples of ERP responses at single electrodes showing the opposite polarity at frontal (Fz) and posterior (Pz) sites. Timecourse of activity elicited by the orthographic stimuli is shown here after it has been subtracted by that in the visual sensory baseline control condition containing no orthographic oddball sequence.

**Figure 3:**
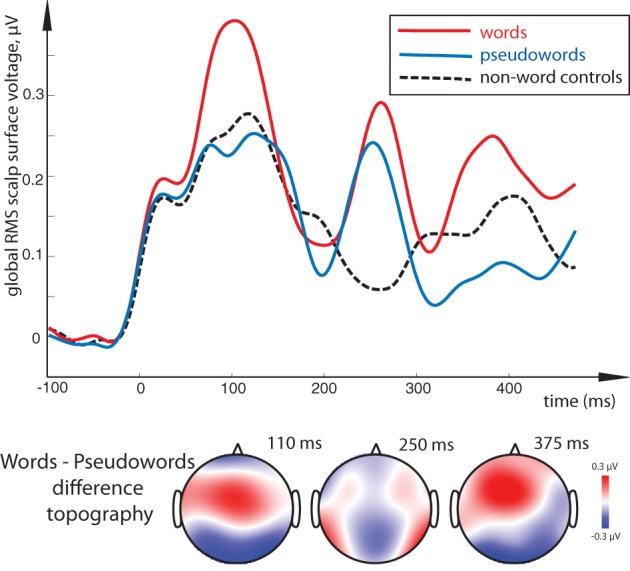
**Lexical familiarity effect: global RMS of responses to word, pseudoword and control non-word stimuli (top) and surface voltage topographic maps for the key word-pseudoword lexicality contrast (bottom).** Lexical familiarity effect (an increased activation for unattended meaningful words as opposed to matched meaningless stimuli) is visible across the three main peaks; it was most significant in the 100–120 ms interval. Remarkably, although words show significant differences from both pseudoword and non-word stimuli, the latter two could not be distinguished statistically. Timecourse of activity elicited by the orthographic stimuli is shown after it has been subtracted by that in the sensory baseline control condition.

All stimulus conditions elicited pronounced ERP responses, with the most prominent peaks in the global response visible at ~110, 250, and ~375 ms (see Figure 2). The first peak exhibited posterior negativity combined with frontal positivity, whereas the reverse—posterior positivity with centro-frontal negativity—was seen for the second peak; the third deflection showed a posterior centro-parietal negativity typical for the N400 time range. Using these overall activity maxima to identify latencies of interest, we then compared window-mean ERP amplitudes at these main activation peaks between different stimuli. Statistical comparison between activation in response to meaningful words as opposed to matched meaningless pseudowords showed a main effect of Lexicality as early as in the first time window (centered at 110 ms), where words produced a significantly stronger response than pseudowords [*F*_(1, 15)_ = 5.76, *p* = 0.03; see Figures 2, 3). This difference was visible as a more negative word deflection at posterior sites [*F*_(1, 15)_ = 5.04, *p* = 0.04], and a more positive one at fronto-central leads [*F*_(1, 15)_ = 5.05, *p* = 0.04]. A non-significant tendency for the same effect could also be observed in the second time window, and, finally, its fully significant rebound took place at the third peak [*F*_(1, 15)_ = 4.93, *p* = 0.04].

A similar difference was revealed by a comparison between words and non-linguistic control stimuli in the first peak [*F*_(1, 15)_ = 9.76, *p* = 0.01] and, although only marginally significant, in the last peak as well [*F*_(1, 15)_ = 3.71, *p* = 0.07]. Interestingly, although visual inspection suggested strong difference between non-word symbols and words also in the second interval (~250 ms), this main effect was not significant when data from the entire electrode array were tested (*p* > 0.7). However, as ANOVA indicated a near-significant interaction between Lexicality and Topography for this contrast [*F*_(4, 16)_ = 2.60, *p* = 0.055], we followed it up with planned comparisons. These showed that the word-non-word difference in this interval was indeed significant but only at the electrodes to the left of the midline [*F*_(1, 15)_ = 4.16, *p* = 0.048] and not at any other sites, likely due to strong between-subject variability in this effect. Pseudowords, in turn, did not differ statistically from the non-linguistic controls in either of the analyzed periods, although visual inspection did suggest a possible discrepancy in the two later intervals.

**Figure 4:**
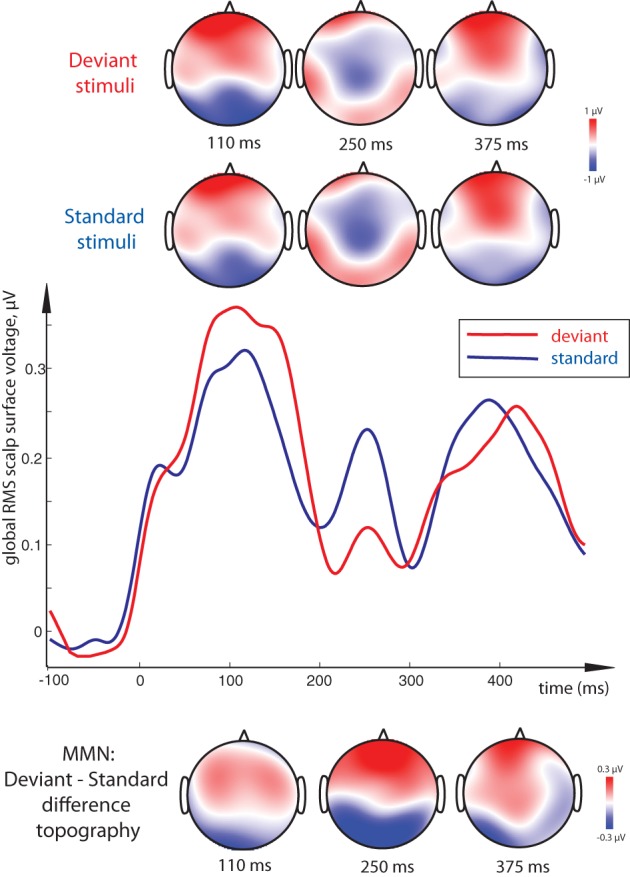
**Visual Mismatch Negativity effect: global RMS of standard and deviant responses to word and pseudoword stimuli (middle), and surface topography of standard and deviant responses (top) and of their difference (vMMN, bottom).** Standard-deviant contrast (vMMN) was most significant at 100–120 ms and 240–260 ms peak intervals, and is seen as a bipolar distribution with posterior negativity and frontal positivity. As the standard-deviant contrast did not interact with lexicality and the vMMN did not distinguish between conditions, data from both word and pseudoword conditions are pooled together for this display. Timecourse of activity elicited by the orthographic stimuli shown here has been subtracted by that in the sensory baseline control condition.

Direct comparison between standard and deviant stimuli revealed a main effect of Stimulus Type, that is, a significant MMN response, with a more negative deviant than standard response at posterior electrodes accompanied by an increased positivity frontally (Figure 4). This contrast was strongly significant in the 100–120 ms time window [*F*_(1, 15)_ = 7.37, *p* = 0.016] as well as in the 240–260 ms one [*F*_(1, 15)_ = 18.51, *p* = 0.001]. Although the latter difference, unlike that in the first peak, could be better described as a posterior *decrease* in positivity and anterior *decrease* in negativity for deviants (amounting to a total decrease in the global RMS curve as well), the net deviant-standard subtraction showed the same relative trend, and the difference topography was thus similar to that in the first peak. In the final time window, no significant mismatch response was found.

Interestingly, whereas we found clear main effects of Lexicality and Stimulus Type, no significant interactions between these factors arose in any of the analysis windows, and vMMN as such did not statistically differ between conditions. Finally, the subjects' performance on the primary behavioral task showed average 85% accuracy indicating good compliance with experimental instructions; mean reaction time was 753 ms.

## Discussion

We recorded ERPs elicited by unattended perifoveally presented meaningful words and orthographically and psycholinguistically matched meaningless pseudowords in a visual oddball sequence, while the subjects were distracted from these materials by a non-linguistic dual feature detection visual task presented in the focus of their attention in the center of the screen. We found (1) an effect of lexicality, i.e., differences in neural responses to words and pseudowords (as well as between words and non-word control stimuli), and (2) an evidence of differential processing of standard vs. deviant stimuli, i.e., the visual correlate of MMN for these lexical stimuli. These effects spanned in time from ~100 to ~400 ms, in line with the previous literature on neural word processing and lexical memory trace activation (Bentin et al., 1999; Martin-Loeches et al., 2005; Hauk et al., 2006; Lau et al., 2008; Carreiras et al., 2009; Pulvermüller et al., 2009). Below, we will discuss these findings in more detail.

### Lexicality effects

The main effect of word-pseudoword difference became exhibited as an *increased word activation* that started very early (from ~100 ms) and, with variable significance, was visible across the response epoch until ~400 ms. As words and pseudowords were matched for orthographic and psycholinguistic features, it is unlikely that it was driven by low-level perceptual differences. Instead, we would like to suggest that this is the lexical familiarity *per se*, i.e., the presence of established memory representations for the meaningful word stimuli, that caused this difference. This is further supported by the remarkable similarity between the present effect and the so-called lexical enhancement in passive *auditory* ERPs. As reviewed in the Introduction, the lexical ERP enhancement has been explained by the activation of a word memory trace in the brain, as opposed to a purely sensory activity for meaningless pseudowords that do not possess memory representations in the brain and thus no corresponding memory trace activation is possible (Shtyrov et al., 2010; MacGregor et al., 2012). In the visual modality, lexical features have been known to affect responses already in 100–160 time range, although those results were obtained for attended and actively processed stimuli (e.g., Ortigue et al., 2004; Hauk et al., 2006), whereas the effect we report here takes place outside of the focus of attention. Previous studies using masked priming paradigm have also found lexico-semantic effects dependent on ‘invisible’ prime words (e.g., Dehaene et al., 2001; Naccache and Dehaene, 2001; Diaz and McCarthy, 2007), albeit their EEG correlates have largely been located in a later time frame, predominantly in the 400 ms range (e.g., Brown and Hagoort, 1993; Kiefer, 2002). At this later time rage, the N400 response typically shows a reduction in amplitude for related prime-probe combinations. Here, we also report a later lexicality effect reaching into ~400 ms time range (in addition to the early differences not typically reported in N400 literature). One important difference between these paradigms, however, is that in the masked priming designs the stimuli usually *are* attended in an active linguistic task (e.g., lexical decision), even though they may escape awareness through masking manipulation. Here, instead, the stimuli are outside the focus of attention while the subjects' task is strictly non-linguistic and does not encourage attentive linguistic processing in any way. Further, while the priming paradigm is typically aimed at revealing relationships between the prime and the probe stimuli, here we are addressing the processing of unattended stimulus *per se* and show that lexical familiarity strongly affects brain responses to such stimuli. Taken together, the current result appears to provide a strong evidence of automatic processing of unattended written language with lexical memory trace activation/access taking place even when this is irrelevant for task requirements and when attention is diverted away from written words. Automatic access to linguistic information in visual modality has been long suggested in behavioral psycholinguistic research (e.g., Glaser and Glaser, 1982; Brown et al., 1995; Naccache and Dehaene, 2001). Here, we show such access neurophysiologically and, furthermore, demonstrate its rapid onset and dynamic timecourse in the brain's activity.

It has been argued that the bases for such automatic lexical activations are distributed neural circuits acting as long-term memory traces for words. Such memory circuits become formed through the process of associative learning in language acquisition, and thus possess strong internal connections that afford memory trace activation automatically, even in the absence of attention (Garagnani et al., 2009; Shtyrov, 2010; Shtyrov et al., 2010). Pseudowords/non-words, on the contrary, do not have such representations, leading to a smaller overall activity under non-attend presentation conditions. Automaticity and rapid speed of lexical activations are likely a consequence of high ecological value and social validity of linguistic communications, which are automatically processed by the brain for any potentially important messages. Previously established in the auditory modality, this automaticity is clearly shown here in the visual modality as well, suggesting similarity in neural word access irrespective of the exact presentation mode.

Although the overall surface topography of the brain responses found here is similar to that known from previous visual studies (e.g., Bentin et al., 1999; Hauk et al., 2006; Lau et al., 2008; Carreiras et al., 2009), exact brain loci of the found automatic lexical familiarity effect cannot be established given the low-resolution EEG method used. For this, future studies are necessary that may employ high-density EEG or/and MEG with neuroanatomically-based source analysis to reveal cortical origins of these lexicality effects. In previous auditory experiments using similar paradigms in fMRI and MEG, these were found in superior- and middle-temporal cortices as well as in inferior-frontal cortex, predominantly in the left hemisphere (Shtyrov et al., 2005, 2008, 2011; Pulvermüller et al., 2006). Further areas, such as the inferior-temporally located visual word-form area as well as angular gyrus, are known to be involved in written word processing (Price, 2001); their involvement in unattended word processing also remains to be addressed in future research.

Interestingly, while the prominent word response around the typical P1/N1 range (~100 ms) here takes the form of a posterior negativity accompanied by frontal positivity, the N170 deflection often found for orthographic materials (e.g., Maurer et al., 2008; Wang et al., 2013) is not obviously present here. There are a few possible explanations for this pattern of results. The most critical difference between this and the earlier visual orthographic studies is the mode of presentation. Rather than presenting the stimuli in the visual focus as it has been conventionally done in N170 studies, we showed them perifoveally where the density of receptors on the retina is reduced (Diaz-Araya and Provis, 1992). Further, the presentation was tachistoscopic, i.e., very brief, which may have also influenced the amplitude of common visual ERPs, including N170. This subtle presentation of the orthographic stimuli was also subject to interference from a massive non-linguistic central stimulus (Figure 1). Alternatively, such a subtle mode of presentation may have also led to a delay in the response peak—this could mean that the deflection at ~250 ms may potentially at least in part be attributed to a weakened and delayed N170. To answer this question with any certainty, future studies will be necessary that will directly compare responses to lexical stimuli using different presentation modes.

### vMMN to orthographic stimuli

In line with previous research into visual MMN (see e.g., Czigler et al., 2002; Pazo-Alvarez et al., 2003; Stagg et al., 2004; Astikainen and Hietanen, 2009; Czigler and Pato, 2009; Kimura et al., 2010; Berti, 2011; Stefanics et al., 2011, 2012), we found that unattended presentation of standard and deviant stimuli in a visual oddball sequence does lead to a vMMN emergence. The current results showed the same relative polarity difference—more negative (or less positive at later times) posterior activity for the deviant than standard stimuli—as that seen with basic visual contrasts in previous vMMN research. The contrasts used in those earlier studies typically included color changes, movement direction, checkerboards and other simple visual objects. Similar to those preivous studies, vMMN seen here occurred early on and took place between 100 and 260 ms, although non-significant effects lasted for longer. The important new finding here is the vMMN elicitation by a subtle orthographic contrast, the change of a single letter in a tachistoscopically presented textual stimulus. This, to our knowledge, is the first demonstration of a vMMN effect for *unattended linguistic materials* suggesting that they are processed automatically early on even when presented outside the foveal attention spot. The only other linguistic vMMN study available to date is a very recent work by Wang et al. (2013), who have shown, using Chinese hieroglyphic characters, vMMN's sensitivity to phonological information. In that study, even though the subjects were not instructed to read the visually presented characters and were instead asked to detect their color, the vMMN was nevertheless strongly influenced by the phonological properties of the stimuli. The important difference between that work and our study is that Wang et al. deviated from the classic vMMN approach, by presenting the stimuli in the focus of visual attention and subjecting them to an explicit behavioral task. In our present work, we have followed more strictly the conventions for visual MMN research by locating the stimuli outside the visual focus of attention and ensuring that the subjects did not perform any stimulus-related activity at all, by distracting them with a spatially distinct primary task. Conceptually, while the current study is focused on automatic lexical effects, the Wang et al. paper deals with automatic extraction of phonological information. The two studies are therefore complementary in various aspects and, together, point toward early automaticity of different types of visual language processing.

While linguistic materials (including vowels, syllables, words and even phrases) have been known to elicit robust auditory MMNs (Pulvermüller and Shtyrov, 2006; Shtyrov and Pulvermüller, 2007), the same is shown here in visual modality, suggesting a certain similarity in linguistic MMN elicitation across modalities. There is, however, an important difference between the previous auditory results and the current visual findings. Auditory MMN research suggested a dominating role of the deviant stimulus's lexical status in eliciting memory trace activation, while reports of lexicality/familiarity effects for frequent standard stimuli have been less consistent (cf. Shtyrov and Pulvermüller, 2002; Jacobsen et al., 2004, 2005). Here, however, we observed no interaction at all between the factors of Lexicality (word vs. pseudoword) and Stimulus Type (standard vs. deviant), and vMMN as such did not statistically differ between conditions. This suggests that, on the one hand, lexical familiarity effects are elicited by standards and deviants alike, and, on the other hand, that vMMN is equally elicited by different stimuli regardless of their lexical familiarity. Given that previous auditory research is not entirely consistent and that the current study is the first foray into the lexical vMMN, it may be premature to discuss whether this difference is due to the modality of presentation, the rigorous within-modality distraction task or possibly some other factors. We would therefore prefer to refrain from addressing this question until further studies using different languages and experimental manipulations are carried out. Similarly, the cortical locus of the lexical vMMN in the brain can only be assessed in future high-density EEG/MEG and possibly fMRI research and cannot be resolved by this first study using a low-resolution EEG methodology.

Finally, application of the vMMN to neurolinguistic processes may open new avenues for this research. Unlike auditorily presented spoken words, visual text does not gradually unfold over time, which allows for stricter control over physical stimulus properties and thus opens a possibility to use a wider range of stimuli. It may also lead to application of linguistic MMN paradigms to situations in which auditory designs are not ideal, such as in noisy environments (e.g., inside an MR scanner) or with hearing-impaired participants, in order to ascertain the degree of automatic linguistic processing in various populations (Shtyrov et al., 2012).

## Conclusions

In a visual oddball sequence, matched short word and pseudoword stimuli were presented tachistoscopically in perifoveal area outside the visual focus of attention, as the subjects' attention was concentrated on a concurrent non-linguistic visual dual task in the center of the screen. Using EEG, we found:

- A visual analogue of the lexical ERP enhancement effect, with unattended written words producing larger brain response amplitudes than matched pseudowords as early as at 100–120 ms;
- A significant visual MMNs at 100-260 ms, here reported for the first time for unattended perifoveally presented lexical stimuli.

The data show a high degree of similarity with earlier auditory research into the neural time course of automatic language processing in the brain. This, in turn, suggests similar or even shared mechanisms of unattended language access in visual and auditory modalities. The current results indicate early and automatic lexical processing of visually presented language in the brain that commences rapidly and may take place outside the focus of visual attention, even under a strong distraction from linguistic input.

### Conflict of interest statement

The authors declare that the research was conducted in the absence of any commercial or financial relationships that could be construed as a potential conflict of interest.

## Footnotes

^1^The distraction from spoken language stimuli is usually implemented by means of a primary visual task, such as watching a film or playing a computer game, although within-modality distraction to contralaterally presented non-speech auditory stimuli has also been successfully used (Pulvermüller et al., 2008).

^2^Stroop effect demonstrates automatic access to the meaning of visually presented word in an experimental task which does not encourage semantic processing or even reading as such. When the name of a color (e.g. “green” or “red”) is printed in a color not denoted by the name (e.g., the word “red” printed in blue ink), color naming takes longer and is more prone to errors than in a non-conflict situation. This and an entire family of similar effects suggest that lexico-semantic information (including individual word semantics) is assessed automatically even though this is not required by the visual task, leading to mutual interference between the two accessed representations (Brown et al., 1995).

^3^Original Cyrillic letters given, with Latin transcription approximating their pronunciation in square brackets.

^4^This is particularly important as some of the pseudowords may have a niche meaning in highly specialized technical vocabularies with a restricted scope of use. As established by the behavioral rankings, the volunteers were not familiar with these stimuli, all of which were thus perceived as meaningless pseudowords.

## References

[B1] Alexandrov A. Boricheva D. Pulvermüller F. Shtyrov Y. (2011). Strength of word-specific neural memory traces assessed electrophysiologically. PLoS ONE 6:e22999 10.1371/journal.pone.0022999 21853063 PMC3154264

[B2] Astikainen P. Hietanen J. K. (2009). Event-related potentials to task-irrelevant changes in facial expressions. Behav. Brain Funct. 5, 30 19619272 10.1186/1744-9081-5-30 PMC2719659

[B3] Bentin S. Mouchetant-Rostaing Y. Giard M. H. Echallier J. F. Pernier J. (1999). ERP manifestations of processing printed words at different psycholinguistic levels: time course and scalp distribution. J. Cogn. Neurosci. 11, 235–260 10.1162/089892999563373 10402254

[B4] Berti S. (2011). The attentional blink demonstrates automatic deviance processing in vision. Neuroreport 22, 664–667 10.1097/WNR.0b013e32834a8990 21841457

[B5] Brown C. Hagoort P. (1993). The processing of the N400: evidence from masked priming. J. Cogn. Neurosci. 5, 34–44 10.1162/jocn.1993.5.1.34 23972118

[B6] Brown T. L. Roos-Gilbert L. Carr T. H. (1995). Automaticity and word perception: evidence from Stroop and Stroop dilution effects. J. Exp. Psychol. Learn. Mem. Cogn. 21, 1395–1411 10.1037/0278-7393.21.6.1395 7490574

[B7] Carreiras M. Gillon-Dowens M. Vergara M. Perea M. (2009). Are vowels and consonants processed differently? Event-related potential evidence with a delayed letter paradigm. J. Cogn. Neurosci. 21, 275–288 10.1162/jocn.2008.21023 18510451

[B8] Czigler I. Balazs L. Winkler I. (2002). Memory-based detection of task-irrelevant visual changes. Psychophysiology 39, 869–873 10.1111/1469-8986.3960869 12462515

[B9] Czigler I. Pato L. (2009). Unnoticed regularity violation elicits change-related brain activity. Biol. Psychol. 80, 339–347 10.1016/j.biopsycho.2008.12.001 19111891

[B10] Czigler I. Weisz J. Winkler I. (2006). ERPs and deviance detection: visual mismatch negativity to repeated visual stimuli. Neurosci Lett. 401, 178–182 10.1016/j.neulet.2006.03.018 16600495

[B11] Dehaene S. Naccache L. Cohen L. Bihan D. L. Mangin J. F. Poline J. B. (2001). Cerebral mechanisms of word masking and unconscious repetition priming. Nat. Neurosci. 4, 752–758 10.1038/89551 11426233

[B12] Diaz M. T. McCarthy G. (2007). Unconscious word processing engages a distributed network of brain regions. J. Cogn. Neurosci. 19, 1768–1775 10.1162/jocn.2007.19.11.1768 17958480

[B13] Diaz-Araya C. M. Provis J. M. (1992). Evidence of photoreceptor migration during early foveal development: a quantitative analysis of human fetal retinae. Vis. Neurosci. 8, 505–514 10.1017/S0952523800005605 1586652

[B14] Friederici A. (2002). Towards a neural basis of auditory sentence processing. Trends Cogn. Sci. 6, 78–84 10.1016/S1364-6613(00)01839-8 15866191

[B15] Garagnani M. Shtyrov Y. Pulvermüller F. (2009). Effects of Attention on what is known and what is not: MEG Evidence for functionally discrete memory circuits. Front. Hum. Neurosci. 3:10 10.3389/neuro.09.010.2009 19680433 PMC2715270

[B16] Glaser M. O. Glaser W. R. (1982). Time course analysis of the Stroop phenomenon. J. Exp. Psychol. Hum. Percept. Perform. 8, 875–894 10.1037/0096-1523.8.6.875 6218237

[B17] Glaser W. R. Glaser M. O. (1989). Context effects in stroop-like word and picture processing. J. Exp. Psychol. Gen. 118, 13–42 10.1037/0096-3445.118.1.13 2522504

[B18] Hagoort P. (2008). The fractionation of spoken language understanding by measuring electrical and magnetic brain signals. Philos. Trans. R. Soc. Lond. B Biol. Sci. 363, 1055–1069 10.1098/rstb.2007.2159 17890190 PMC2606796

[B19] Hauk O. Davis M. H. Ford M. Pulvermüller F. Marslen-Wilson W. D. (2006). The time course of visual word recognition as revealed by linear regression analysis of ERP data. Neuroimage 30, 1383–1400 10.1016/j.neuroimage.2005.11.048 16460964

[B20] Henson R. N. (2003). Neuroimaging studies of priming. Prog. Neurobiol. 70, 53–81 10.1016/S0301-0082(03)00086-8 12927334

[B21] Heslenfeld D. J. (2003). Visual mismatch negativity, in Detection of Change: Event-Related Potential and fMRI Findings, ed Polich J. (Boston, MA: Kluver Academic Press), 41–59

[B22] Jacobsen T. Horvath J. Schroger E. Lattner S. Widmann A. Winkler I. (2004). Pre-attentive auditory processing of lexicality. Brain Lang. 88, 54–67 10.1016/S0093-934X(03)00156-1 14698731

[B23] Jacobsen T. Schroger E. Winkler I. Horvath J. (2005). Familiarity affects the processing of task-irrelevant auditory deviance. J. Cogn. Neurosci. 17, 1704–1713 10.1162/089892905774589262 16269107

[B24] Kiefer M. (2002). The N400 is modulated by unconsciously perceived masked words: further evidence for an automatic spreading activation account of N400 priming effects. Brain Res. Cogn. Brain Res. 13, 27–39 10.1016/S0926-6410(01)00085-4 11867248

[B25] Kimura M. Ohira H. Schroger E. (2010). Localizing sensory and cognitive systems for pre-attentive visual deviance detection: an sLORETA analysis of the data of Kimura et al. (2009). Neurosci. Lett. 485, 198–203 10.1016/j.neulet.2010.09.011 20849925

[B26] Korpilahti P. Krause C. M. Holopainen I. Lang A. H. (2001). Early and late mismatch negativity elicited by words and speech-like stimuli in children. Brain Lang. 76, 332–339 10.1006/brln.2000.2426 11247648

[B27] Lau E. F. Phillips C. Poeppel D. (2008). A cortical network for semantics: (de)constructing the N400. Nat. Rev. Neurosci. 9, 920–933 10.1038/nrn2532 19020511

[B28] Lee C. Y. Liu Y. N. Tsai J. L. (2012). The time course of contextual effects on visual word recognition. Front. Psychol. 3:285 10.3389/fpsyg.2012.00285 22934087 PMC3422729

[B29] MacGregor L. J. Pulvermuller F. van Casteren M. Shtyrov Y. (2012). Ultra-rapid access to words in the brain. Nat. Commun. 3, 711 22426232 10.1038/ncomms1715 PMC3543931

[B30] Martin-Loeches M. Sommer W. Hinojosa J. A. (2005). ERP components reflecting stimulus identification: contrasting the recognition potential and the early repetition effect (N250r). Int. J. Psychophysiol. 55, 113–125 10.1016/j.ijpsycho.2004.06.007 15598521

[B31] Maurer U. Rossion B. McCandliss B. D. (2008). Category specificity in early perception: face and word n170 responses differ in both lateralization and habituation properties. Front. Hum. Neurosci. 2:18 10.3389/neuro.09.018.2008 19129939 PMC2614860

[B32] Näätänen R. Paavilainen P. Rinne T. Alho K. (2007). The mismatch negativity (MMN) in basic research of central auditory processing: a review. Clin Neurophysiol. 118, 2544–2590 10.1016/j.clinph.2007.04.026 17931964

[B33] Naccache L. Dehaene S. (2001). Unconscious semantic priming extends to novel unseen stimuli. Cognition 80, 215–229 10.1016/S0010-0277(00)00139-6 11274983

[B34] Oken B. S. Chiappa K. H. Gill E. (1987). Normal temporal variability of the P100. Electroencephalogr. Clin. Neurophysiol. 68, 153–156 10.1016/0168-5597(87)90042-6 2435531

[B35] Oldfield R. C. (1971). The assessment and analysis of handedness: the Edinburgh Inventory. Neuropsychologia 9, 97–113 10.1016/0028-3932(71)90067-4 5146491

[B36] Ortigue S. Michel C. M. Murray M. M. Mohr C. Carbonnel S. Landis T. (2004). Electrical neuroimaging reveals early generator modulation to emotional words. Neuroimage 21, 1242–1251 10.1016/j.neuroimage.2003.11.007 15050552

[B37] Pazo-Alvarez P. Cadaveira F. Amenedo E. (2003). MMN in the visual modality: a review. Biol. Psychol. 63, 199–236 10.1016/S0301-0511(03)00049-8 12853168

[B38] Pettigrew C. M. Murdoch B. M. Chenery H. J. Kei J. (2004). The relationship between the mismatch negativity (MMN) and psycholinguistic models of spoken word processing. Aphasiology 18, 3–28

[B39] Price C. J. (2001). Functional-imaging studies of the 19th Century neurological model of language. Rev. Neurol. 157, 833–836 11677405

[B40] Pulvermüller F. Shtyrov Y. (2006). Language outside the focus of attention: the mismatch negativity as a tool for studying higher cognitive processes. Prog. Neurobiol. 79, 49–71 10.1016/j.pneurobio.2006.04.004 16814448

[B41] Pulvermüller F. Shtyrov Y. Hasting A. S. Carlyon R. P. (2008). Syntax as a reflex: neurophysiological evidence for early automaticity of grammatical processing. Brain Lang. 104, 244–253 10.1016/j.bandl.2007.05.002 17624417

[B42] Pulvermüller F. Shtyrov Y. Hauk O. (2009). Understanding in an instant: neurophysiological evidence for mechanistic language circuits in the brain. Brain Lang. 110, 81–94 10.1016/j.bandl.2008.12.001 19664815 PMC2734884

[B43] Pulvermüller F. Shtyrov Y. Ilmoniemi R. J. (2005). Brain signatures of meaning access in action word recognition. J. Cogn. Neurosci. 17, 884–892 10.1162/0898929054021111 15969907

[B44] Pulvermüller F. Shtyrov Y. Ilmoniemi R. J. Marslen-Wilson W. D. (2006). Tracking speech comprehension in space and time. Neuroimage 31, 1297–1305 10.1016/j.neuroimage.2006.01.030 16556504

[B45] Sharoff S. (2001). Word frequency dictionary of Russian language. Russ. Res. Inst. Artif. Intellect. http://www.artint.ru/projects/frqlist.php

[B46] Shtyrov Y. (2010). Automaticity and attentional control in spoken language processing: neurophysiological evidence. Ment. Lexicon 5, 255–276 10.1075/ml.5.2.06sht

[B47] Shtyrov Y. Hauk O. Pulvermüller F. (2004). Distributed neuronal networks encoding category-specific semantic information as shown by the mismatch negativity to action words. Eur. J. Neurosci. 19, 1083–1092 10.1111/j.0953-816X.2004.03126.x 15009156

[B48] Shtyrov Y. Kujala T. Pulvermüller F. (2010). Interactions between language and attention systems: early automatic lexical processing? J. Cogn. Neurosci. 22, 1465–1478 10.1162/jocn.2009.21292 19580394

[B49] Shtyrov Y. Osswald K. Pulvermüller F. (2008). Memory traces for spoken words in the brain as revealed by the hemodynamic correlate of the mismatch negativity. Cereb. Cortex 18, 29–37 10.1093/cercor/bhm028 17412721

[B50] Shtyrov Y. Pihko E. Pulvermüller F. (2005). Determinants of dominance: is language laterality explained by physical or linguistic features of speech? Neuroimage 27, 37–47 16023039 10.1016/j.neuroimage.2005.02.003

[B51] Shtyrov Y. Pulvermüller F. (2002). Neurophysiological evidence of memory traces for words in the human brain. Neuroreport 13, 521–525 10.1097/00001756-200203250-00033 11930174

[B52] Shtyrov Y. Pulvermüller F. (2007). Language in the mismatch negativity design: motivations, benefits and prospects. J. Psychophysiol. 21, 176–187 10.1027/0269-8803.21.34.176

[B53] Shtyrov Y. Smith M. Horner A. Henson R. Bullmore E. Nathan P. (2012). Attention to language: novel MEG paradigm for registering involuntary language processing in the brain. Neuropsychologia 50, 2605–2616 10.1016/j.neuropsychologia.2012.07.012 22820635 PMC3657698

[B54] Shtyrov Y. Kimppa L. Pulvermüller F. Kujala T. (2011). Event-related potentials reflecting the frequency of unattended spoken words: a neuronal index of connection strength in lexical memory circuits? Neuroimage 55, 658–668 21146619 10.1016/j.neuroimage.2010.12.002

[B55] Stagg C. Hindley P. Tales A. Butler S. (2004). Visual mismatch negativity: the detection of stimulus change. Neuroreport 15, 659–663 10.1097/00001756-200403220-00017 15094471

[B56] Stefanics G. Csukly G. Komlosi S. Czobor P. Czigler I. (2012). Processing of unattended facial emotions: a visual mismatch negativity study. Neuroimage 59, 3042–3049 10.1016/j.neuroimage.2011.10.041 22037000

[B57] Stefanics G. Kimura M. Czigler I. (2011). Visual mismatch negativity reveals automatic detection of sequential regularity violation. Front. Hum. Neurosci. 5:46 10.3389/fnhum.2011.00046 21629766 PMC3099311

[B58] Wang X. D. Liu A. P. Wu Y. Y. Wang P. (2013). Rapid extraction of lexical tone phonology in Chinese characters: a visual mismatch negativity study. PLoS ONE 8:e56778 10.1371/journal.pone.0056778 23437235 PMC3577723

